# Orthodontics and Endodontics Clinical Practice Correlation: A Narrative Review

**DOI:** 10.7759/cureus.56821

**Published:** 2024-03-24

**Authors:** AbdulMajeed A AlMogbel, Shatha Alasmary, Shaden Alfarraj, Raya Alenazi, Rawan Albuti

**Affiliations:** 1 Department of Orthodontics and Pediatric Dentistry, College of Dentistry, Qassim University, Buraydah, SAU; 2 Department of Dentistry, King Saud Bin Abdulaziz University for Health Sciences, College of Dentistry, Riyadh, SAU

**Keywords:** tooth movement, management, traumatic teeth, endodontics, orthodontics, evidence-based dentistry, dentistry

## Abstract

Research on the connection between endodontic therapy and orthodontics is lacking. This overview of the literature synthesizes the findings from the fields of orthodontics and endodontics and explains how they are related. Beginning with the diagnosis, treating the patient at the appropriate time, moving endodontically treated teeth and traumatized teeth, resorbing roots by orthodontic mechanics, and managing traumatized teeth with orthodontic therapy.

Multiple electronic databases were utilized including (PubMed, Scopus, Science Direct, and Web of Science) to perform manual literature searches. A total of 31 articles were reviewed and summarized in this paper in keywords like "Endodontically Treated Teeth and Orthodontic Treatment," "Endodontically Treated Tooth and Orthodontic Movement," "Orthodontic Treatment in RCT Teeth," "Root Canal Treatment with Orthodontic Movement," "Trauma with Orthodontic Movement," "Orthodontic and Endodontic."

Orthodontic treatment of endodontically treated and traumatized teeth is a subject of controversy. The lack of research on the topic makes it a hard decision to make when to treat these teeth. Especially given that both orthodontic and endodontic treatments have multiple consequences on each other’s outcomes. Thus, it is crucial for clinicians to understand how they integrate and have a guideline to refer to during decision-making.

Successful orthodontic tooth movement could be carried out immediately after endodontic treatment. However, traumatized teeth need a follow-up period before initiating orthodontic movement, which ranges from three months to 12 months depending on the type of trauma and severity. Careful radiographic and clinical follow-up should be done during the healing period. Collaborative teamwork is important between orthodontists and endodontists for the success of treatment, and to achieve satisfactory outcomes.

## Introduction and background

In the fast-paced world of modern dentistry, accurate diagnosis is key to effective treatment and patient care. One area that has gained particular attention in recent years is the field of orthodontics and endodontics diagnosis, and how both diagnoses can influence their treatment outcome. Understanding the complex relationship between endodontics and orthodontics is essential for dental professionals seeking to provide comprehensive dental care. Orthodontic tooth movement could have multiple consequences on dental pulp. Studies have shown that orthodontic treatment could have a transient inflammatory response on dental pulp [1], external root resorption [2], and short-term dental pulp sensitivity [3], all of which and more should be considered before initiation of orthodontic tooth movement. Multiple factors influence the occurrence of complications during orthodontic tooth movement, such as forces used during movement of teeth [4]. It has been proven that heavy force causes external root resorption, more than the teeth that were subjected to light forces [4]. Also, the presence of endodontically treated teeth and their pulp vitality and periapical diagnosis before the root canal treatment plays a role in the planning and timing of orthodontic treatment [5]. And if teeth were traumatized before initiation of orthodontic tooth movement [6]. By understanding how these two disciplines interact, dental practitioners can optimize treatment plans and achieve better outcomes for their patients. In this review, we will go into the intricacies of endo-ortho diagnosis, exploring the principles.

## Review

A manual literature search was conducted using a number of electronic databases, including PubMed, Scopus, Science Direct, and Web of Science. The present paper reviews and summarizes thirty-one articles using terms such as "Orthodontic and Endodontic," "Orthodontic and Endodontic Movement," "Orthodontic Treatment in RCT Teeth," "Root Canal Treatment with Orthodontic Movement," "Trauma with Orthodontic Movement," and "Orthodontic and Endodontic Dentistry."

Orthodontics and endodontics relationship and clinical diagnosis

An essential component of dental care is understanding the interaction between endodontic and orthodontic therapy. For dental practitioners to give their patients thorough and efficient care, they must comprehend this relationship. The possibility that the results of endodontic and orthodontic treatments may affect one another is one of the main justifications for understanding their interaction. For instance, tooth movement during orthodontics may have an impact on the dental pulp's health and vitality [7]. This is especially true for teeth with completed apical formation, where orthodontic tooth movement can cause degenerative and inflammatory responses in the pulp [7]. The neurovascular system releases particular neurotransmitters called neuropeptides. Those can affect both blood flow and cellular metabolism of the pulp. Which in turn can influence tooth movement [7]. The influence is in the beginning and the continuation of apical remodeling which happens during orthodontic tooth movement [7]. A history of past or continuous pulpal injury or caries has a major role in determining the severity of these changes [7]. Coordinating the timing of treatments has a major influence on treatment outcomes, such as completing the root canal treatment before initiating orthodontic tooth movement and ensuring periapical healing in case of periapical pathosis [8]. Orthodontic tooth movement can be started as soon as six months after completion of root canal treatment given that the treatment outcome is satisfactory in both aspects clinically and radiographically. As well as the tooth being asymptomatic [8]. A follow-up period of six months after initiation of orthodontic movement should be done to ensure the absence of complications [8]. 

The healing of the apical periodontal ligament is an important consideration when deciding when to initiate orthodontic movement after endodontic treatment. The apical periodontal ligament is responsible for providing support and stability to the tooth [9]. After endodontic treatment, the apical periodontal ligament undergoes a healing process [9]. During this healing process, it is important to allow sufficient time for the ligament to fully recover and regain its strength before subjecting the tooth to orthodontic forces [9]. Studies have shown that the healing period of the apical periodontal ligament can range from a few weeks to several months, depending on various factors such as the complexity of the endodontic treatment and the individual patient's healing capacity [9].

Other studies suggest the healing period for periapical lesions ranges from six to 12 months after completion of root canal treatment to ensure complete healing [10]. And even after 12 months signs of complete healing might not be present, and complete healing may require up to four years [10]. One study by Delivanis and Sauer investigated the effect of orthodontic forces on teeth with recent endodontic treatment [11]. They found that when orthodontic forces were applied immediately after endodontic treatment, there was a higher risk of root resorption compared to when a healing period was allowed [11]. The study concluded that it is crucial to give the apical periodontal ligament enough time to heal before initiating orthodontic movement to minimize the risk of root resorption [11]. The potential for root resorption should also be taken into account when determining the timing of orthodontic movement after endodontic treatment. Studies have indicated that teeth with previous root canal treatment exhibit a lower propensity for apical root resorption during orthodontic tooth movement compared to teeth without endodontic treatment [12]. The Removal of pulpal tissue will lead to loss of neuropeptide release which in turn results in a decrease in calcitonin gene-related peptide immunoreactive (CGRP-IR) fibers which play a role in external root resorption [12]. All of what was previously mentioned necessitates the importance of collaboration between orthodontists and endodontists in the assessment of teeth before initiation of orthodontic tooth movement, during orthodontic treatment in case of developing signs and symptoms of external root resorption, and after in case of external root resorption or caries that progress into pulpal pathoses.

Orthodontic movement of endodontically treated teeth consideration 

Periapical disease refers to an inflammatory condition that affects the tissues surrounding the apex of a tooth's root [13,14]. This condition typically arises due to a bacterial infection that originates from the dental pulp [13,14]. In the literature, it was found that periodontal disease is a common complication of periapical disease [14]. This finding highlights the interplay between these two conditions and raises questions about their relationship to orthodontic treatment.

In the context of periapical disease, orthodontic movement of teeth may have implications for the pre-existing lesions. While orthodontic treatment has been shown to have limited effects on the healing process of periapical lesions, it is important to acknowledge that complications can arise [15]. Complications associated with orthodontic treatment on endodontically treated teeth include apical root resorption and periodontal problems [16]. However, it should be noted that these complications are relatively rare and can be managed effectively with proper treatment protocols [16]. In the case of vital teeth, they may be more susceptible to root resorption compared to pulp-less teeth [17]. Various treatment approaches are available for periapical disease, depending on the severity and extent of the lesions. These approaches include root canal therapy, apical surgery, and regenerative techniques such as guided tissue regeneration [18-20]. Root canal therapy is the most common and effective treatment for periapical disease [18]. The infected pulp is removed from the root canal, and the canal is then cleaned, shaped, and filled with a biocompatible material to prevent reinfection [18]. Apical surgery, also known as periapical surgery or apicoectomy, is performed when root canal therapy alone is insufficient to eliminate the infection [19]. During apical surgery, the apex of the tooth is accessed, and any infected or damaged tissue is removed [19]. The remaining root tip is then sealed to prevent further infection [19]. Regenerative techniques such as guided tissue regeneration involve the use of barrier membranes and growth factors to encourage the regeneration of damaged periapical tissues [20]. These techniques aim to promote the regrowth of bone and periodontal tissues in the periapical region. Overall, the literature highlights the importance of accurately diagnosing and treating periapical disease before initiating orthodontic treatment [20]. This can help minimize the risk of exacerbating existing periapical lesions and prevent complications such as root resorption [20]. 

To minimize the risk of periapical disease in orthodontic patients, several preventive measures can be implemented. These measures include a thorough examination and assessment of the periapical region before initiating orthodontic treatment to identify any pre-existing lesions or signs of disease [21]. Also, proper oral hygiene education and reinforcement throughout orthodontic treatment prevent the accumulation of plaque and bacteria, which can contribute to periapical inflammation and infection [22]. Additionally, regular monitoring of the periapical region during orthodontic treatment through radiographic evaluations to detect any changes or progression of existing periapical lesions [23]. Avoiding excessive forces during orthodontic treatment to minimize the risk of inflammation and trauma to periapical tissues [16]. Lastly, collaboration between orthodontists and endodontists ensures comprehensive care for patients with periapical disease undergoing orthodontic treatment. This collaborative approach allows for timely referral for endodontic treatment if periapical disease is present or suspected and enables effective communication and coordination of treatment to prevent the exacerbation of periapical disease during orthodontic movement [21]. The periapical disease can have significant implications for orthodontic treatment [23]. Orthodontists should be aware of the potential risks and take preventive measures to minimize the impact on periapical health [23]. This includes conducting thorough examinations, promoting proper oral hygiene, monitoring the periapical region during treatment, applying controlled force, and collaborating with endodontists to provide comprehensive care [16,21-23]. In conclusion, periapical disease can have an impact on orthodontic treatment due to the potential for increased inflammatory reactions, root resorption, and delayed healing [23]. Table 1 is a summary of the type of endodontic treatment and the suggested time to start or resume orthodontic teeth movement.

**Table 1:** Types of endodontic treatment and the time to start orthodontic teeth movement

Type of endodontic treatment	Timing to resume orthodontic treatment
Root canal treated teeth due to caries	If no periapical pathosis, immediate orthodontic movement could be done [7].
Root canal treated teeth with periapical lesion	Six to 12 months after endodontic treatment [10].
Apicoectomy	Not enough data was found in the literature. A case report suggested a six-month recall to determine when orthodontic treatment should start [24].

Root resorption and endodontic sequelae of orthodontic treatment

Orthodontically induced root inflammatory resorption is a common complication that occurs during and after orthodontic treatment [25]. It refers to the pathological process of root structure loss due to excessive mechanical forces applied during tooth movement [25].

Brezniak and Wasserstein coined the term "orthodontically induced inflammatory root resorption" to distinguish this type of root resorption from other types caused by trauma, periapical lesions, or periodontal disease [26]. According to studies, it was found that approximately one in every 20 individuals undergoing orthodontic treatment experienced root resorption of at least 5 mm [27]. This highlights the significant impact of orthodontically induced inflammatory root resorption on patients receiving orthodontic therapy [27]. While orthodontically induced root resorption can occur in any tooth, it is most commonly observed in the anterior teeth, specifically the maxillary incisors [28]. Multiple factors have been proposed to contribute to root resorption after orthodontic treatment. These factors can be grouped into two main categories: orthodontic treatment factors and patient-related factors [28,29]. Orthodontic treatment factors include the length of treatment, type of tooth movement, and the amount of force applied during orthodontic treatment [28]. Some sources suggest that heavy orthodontic forces can increase the risk of root resorption [16,28]. Patient-related factors include individual variations in root morphology, genetic predisposition, and systemic conditions such as hormonal imbalance or certain medications that may affect root resorption [29]. Additionally, age, previous dental trauma, and allergies have also been identified as potential risk factors for root resorption [28]. There is no definitive treatment for orthodontically induced root resorption. Treatment approaches for orthodontically induced root resorption mainly focus on monitoring and managing the condition rather than providing a cure. Studies have suggested that early detection and intervention may help to minimize the progression of root resorption [23]. One approach to treatment is regular monitoring of the resorption process through radiographic examinations [23]. These examinations can help track the progression of root resorption and determine if any changes need to be made to the orthodontic treatment plan [23]. Another approach to treatment is reducing the magnitude of orthodontic force applied to the teeth [16]. This can be achieved by using lighter forces, shorter treatment durations, or a combination of both [16]. Additional strategies include the use of low-friction orthodontic systems and the incorporation of temporary anchorage devices to minimize the forces applied to the teeth [30].

Some key findings and recommendations from the literature include regular radiographic examinations are essential for monitoring the progress of root resorption during orthodontic treatment. Also, the use of lighter forces and shorter treatment durations may help reduce the risk of orthodontically induced root resorption [16]. Multidisciplinary collaboration between orthodontists and endodontists is crucial for the effective management of orthodontically induced root resorption [21]. The incorporation of low-friction orthodontic systems and temporary anchorage devices can help minimize the forces applied to the teeth, thereby reducing the risk of root resorption [30]. To conclude, the treatment of orthodontically induced external root resorption is a complex issue in orthodontics. Further research is needed to fully understand the underlying mechanisms and develop targeted treatment strategies.

Trauma and orthodontic management

There is a consensus among experts that the timing of orthodontic tooth movement in traumatized teeth should be carefully considered. Research has shown that the timing of orthodontic tooth movement in traumatized teeth should be approached with caution and careful consideration [6]. It is also important to note that the use of custom-made mouthguards during orthodontic treatment can help prevent dental trauma [6]. Overall, the literature on trauma and orthodontic management highlights the need for careful assessment, monitoring, and interdisciplinary collaboration in treating patients with dental trauma [6,7]. Orthodontic management involves assessing and managing the risk of root resorption in traumatized teeth during orthodontic treatment [7]. Previously traumatized teeth undergoing orthodontic tooth movement can cause root resorption [6]. Therefore, a detailed assessment of the trauma history and thorough clinical and radiographic examinations are essential before initiating orthodontic treatment [7]. This process will help identify teeth at a high risk of root resorption and allow for appropriate treatment planning and patient education [7]. Emphasis should be made on the importance of close collaboration between orthodontists and endodontists in the detailed assessment and collaborative management of trauma in pre-orthodontic patients. By working together, they can ensure that the safe orthodontic treatment plan takes into account the long-term prognosis of traumatized teeth and minimizes the risk of further complications. 

In cases where there is evidence of root resorption or other severe structural damage, orthodontic treatment goals may need to be revised [7]. Teeth with these characteristics are at a higher risk of experiencing further resorption during treatment [7]. It is important to note that there are limitations to current research in this field. Most studies consist mainly of anecdotal case reports or small-scale reviews published prior to 1999. While resources exist for managing dental injuries, this paper aims to provide a summary of the most important clinical practice data rather than an in-depth discussion. The literature also emphasizes accurate diagnoses, appropriate treatment timing, and collaborative efforts among orthodontists, and endodontists. Table 2 provides a brief summary of the types of dental trauma for orthodontic treatment along with the correct timing to move or resume traumatized teeth.

**Table 2:** Types of trauma and time to start tooth movement

Type of trauma	Timing to resume orthodontic treatment
Concussion, subluxation, extrusion	Three months (Kindelan et al., 2008) [6].
Avulsion and re-implantation intrusion	One year if ankylosis is not detected (Kindelan et al., 2008) [6].
Luxation injuries	Postpone treatment for three to six months sometimes up to 12 months in case of severe trauma (Patel et al., 2022) [31]. One year if ankylosis is not detected (Kindelan et al., 2008) [6].
Crown and crown/root fracture (complicated/ uncomplicated)	Three months (Kindelan et al., 2008) [6]. The observation period before orthodontic treatment is three months (Sandler et al., 2019) [32].
Root fracture	Observation for one to two years, or shorter, if asymptomatic.Teeth should not be moved until successful endodontic treatment and connective tissue healing of the coronal fragment has occurred (Sandler et al., 2019) [32].
Auto transplanted teeth	Two to three months, after periodontal ligament healing which takes place in eight months (Kindelan et al., 2008) [6].
Orthodontic management of teeth with Infection-related resorption due to trauma	At least one year; orthodontic movement should only start once the infection is under control with stable results (Sandler et al., 2019) [32].
Orthodontic management of teeth with regenerative endodontic/revitalization due to trauma	It is advisable to delay orthodontic treatment until stable final results have been observed with a minimum review period of two years (Sandler et al., 2019) [32].
Root canal treated teeth, due to trauma, obturated with gutta percha	One year, in a mature closed apex tooth (Kindelan et al., 2008) [6].
Trauma during orthodontic treatment	Although there is no evidence to provide the clinician with definitive answers, the prudent clinician will reassess the case after the traumatic injury. Depending on the extent of the injury and the current stage of treatment (beginning, middle, and finishing), the clinician may elect to discontinue treatment, modify treatment, or finish as planned [6].

## Conclusions

Orthodontics and endodontics are two specialized fields in dentistry that focus on different aspects of oral health. Orthodontics primarily deals with the alignment and positioning of teeth, while endodontics focuses on treating diseases and issues that affect the dental pulp and root canal. However, these two fields are not mutually exclusive and often have a relationship that can benefit patients. Orthodontic treatment may be necessary before or after endodontic procedures to ensure proper alignment and stability of the teeth. The success of endodontic treatment can be influenced by orthodontic factors such as tooth position and occlusion. Effective communication and collaboration between orthodontists and endodontists are crucial for providing optimal care to patients. It is important for orthodontists and endodontists to work together to provide comprehensive and coordinated care. This can lead to better treatment outcomes, improved patient satisfaction, and overall oral health.
